# Novel Nanocomposites Based on Functionalized Magnetic Nanoparticles and Polyacrylamide: Preparation and Complex Characterization

**DOI:** 10.3390/nano9101384

**Published:** 2019-09-27

**Authors:** Eugenia Tanasa, Catalin Zaharia, Ionut-Cristian Radu, Vasile-Adrian Surdu, Bogdan Stefan Vasile, Celina-Maria Damian, Ecaterina Andronescu

**Affiliations:** 1 University Politehnica of Bucharest, Faculty of Applied Chemistry and Materials Science, 060042 Bucharest, Romania; eugenia.vasile27@gmail.com (E.T.); adrian.surdu@upb.ro (V.-A.S.); bogdan.vasile@upb.ro (B.S.V.); ecaterina.andronescu@upb.ro (E.A.); 2 National Centre for Micro and Nanomaterials, University Politehnica of Bucharest, 060042 Bucharest, Romania; 3 Advanced Polymer Materials Group, University Politehnica of Bucharest, 060042 Bucharest, Romania; radu.ionucristian@gmail.com (I.-C.R.); celina.damian@yahoo.com (C.-M.D.); 4 National Research Center for Food Safety, University Politehnica of Bucharest, 060042 Bucharest, Romania

**Keywords:** magnetic nanoparticles, polyacrylamide, functionalization, nanocomposite, hydrogel

## Abstract

This paper reports the synthesis and complex characterization of nanocomposite hydrogels based on polyacrylamide and functionalized magnetite nanoparticles. Magnetic nanoparticles were functionalized with double bonds by 3-trimethoxysilyl propyl methacrylate. Nanocomposite hydrogels were prepared by radical polymerization of acrylamide monomer and double bond modified magnetite nanoparticles. XPS spectra for magnetite and modified magnetite were recorded to evaluate the covalent bonding of silane modifying agent. Swelling measurements in saline solution were performed to evaluate the behavior of these hydrogels having various compositions. Mechanical properties were evaluated by dynamic rheological analysis for elastic modulus and vibrating sample magnetometry was used to investigate the magnetic properties. Morphology, geometrical evaluation (size and shape) of nanostructural characteristics and the crystalline structure of the samples were investigated by SEM, HR-TEM and selected area electron diffraction (SAED). The nanocomposite hydrogels will be further tested for the soft tissue engineering field as repairing scaffolds, due to their mechanical and magnetization behavior that can stimulate tissue regeneration.

## 1. Introduction

Polymeric hydrogel-like materials are a category of soft materials containing crosslinked hydrophilic networks with a high swelling ability. The hydrophilic nature of the macromolecular chains is based, in general, on side hydrophilic active groups [1,2,3,4]. The cross-linking reaction of hydrophilic chains is an absolute requirement for dissolution avoiding of polymeric material. The generation of a cross-linked network assumes formation of inter and intramolecular bridges, which do not allow the solvent molecules to solve and unfold the macromolecules. Thus, the solvent can only penetrate among polymeric molecules and swell the material [5,6]. In the swollen state, the polymeric hydrogel exhibits brittleness and obvious low mechanical properties. These disadvantages seriously limit their usage in special biomedical applications. The use of polymeric hydrogels is directly related to the intrinsic mechanical properties in the swollen state. A relatively new concept of polymeric nanocomposite hydrogels has started to overcome these problems by combining the advantage of polymeric hydrogels with the advantage of polymeric nanocomposites [7,8,9,10,11,12,13,14,15]. Nanocomposite hydrogels have been developed by various methods, such as in situ polymerization or pre-modified inorganic nanoparticles [16,17,18,19,20,21]. Modified inorganic nanomaterials have gained special attention, as they can be used as inorganic crosslinkers. These types of modified crosslinkers exhibit a unique flexible intrinsic structure with a serious contribution to improving mechanical properties [22]. The major limitation of the swollen hydrogels is related to the network generation process based on traditionally low molecular weight organic crosslinkers. The limitation of classic organic crosslinkers, due to their relative low number of available groups for reactions with polymeric chains, can be overcome by inorganic nanoparticles modified with multiple groups. A suitable modification involves designing molecular architectures with long and short intermolecular and intramolecular bridges at the same time [7,23,24,25,26,27]. The mechanical stress generates the fracture first of short chains to partially dissipate the elastic energy, while the long chains take the remaining loading. Meanwhile, the hydrogel is still intact [4,28,29,30,31]. Inorganic nanoparticles as crosslinkers possess high stretchability, elasticity and superior toughness for polymeric nanocomposite hydrogels, with potential use in soft tissue applications. Inorganic nanoparticles such as magnetite exhibit a high potential for modification with functional groups, due to the presence of hydroxyl groups. They show outstanding physico-chemical properties due to the presence of both species of iron [32,33,34]. Furthermore, magnetite has been used with great success for various biomedical applications [34,35,36,37], including cellular imaging [38] or cancer diagnosis, monitoring and treatment [39]. 

This research study is focused on the development of nanocomposite networks crosslinked by highly-functionality modified magnetite with enhanced stretchability and elasticity for biological tissue applications.

## 2. Materials and Methods 

### 2.1. Materials

The reagents used for the synthesis of the magnetic iron oxide nanoparticles were iron chloride iron (III) chloride (FeCl_3_, 97%), ferrous sulfate heptahydrate (FeSO_4_·7H_2_O) and ammonium hydroxide solution (NH_4_OH). The acrylamide monomer, 3-trimethoxysilyl propyl methacrylate modifier agent and potassium persulfate initiator were used for the preparation of hydrogels. All the reagents were supplied by Sigma-Aldrich, 3050 Spruce Street, St. Louis, MO, United States.

### 2.2. Synthesis of Magnetite (Fe_3_O_4_) Nanoparticles

The synthesis of the Fe_3_O_4_ nanoparticles (MNPs) was carried out at room temperature, by co-precipitation method, starting from iron (III) chloride, ferrous sulfate heptahydrate and ammonium hydroxide solution [40,41]. The iron chloride was dissolved in deionized water to give a clear solution. Under vigorous magnetic stirring, the FeSO_4_·7H_2_O was added to the solution (Fe^2+^/Fe^3+^ = 1:2 molar ratio). Independently, an aqueous solution of ammonium hydroxide is prepared, and the mixture solution resulting from the iron chloride and ferrous sulfate heptahydrate was added to it. Magnetite nanoparticles formed and precipitated. The MNPs were separated from the reaction medium using a strong magnet. The powder was rinsed several times with distilled water until reaching a neutral pH (pH = 7) in the washing solution. After washing, the precipitate was dried for 12h in air oven, at 60 °C.

### 2.3. Synthesis of Double Bond Modified Magnetite Nanoparticles

**Figure 1:**
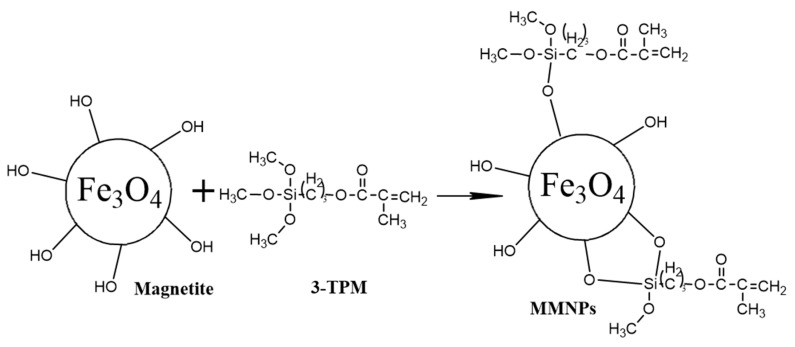
Modification of magnetite nanoparticles with double bonds.

The surface modification of the magnetic nanoparticles with double bonds was carried out in several steps, as follows (Figure 1). Briefly, 2 g of MNPs were reacted with 4 mL of 3-trimethoxysilyl propyl methacrylate (3-TPM) by dispersion in 40 mL of toluene for 24 hours at room temperature under magnetic stirring. The modified magnetic nanoparticles (denoted by MMNPs) were then washed several times with toluene to remove the unmodified MNPs and unreacted 3-TPM by centrifugation and then dried.

### 2.4. Preparation of Polyacrylamide/MMNPs Nanocomposite Hydrogels (PAA/MMNPs)

**Figure 2:**
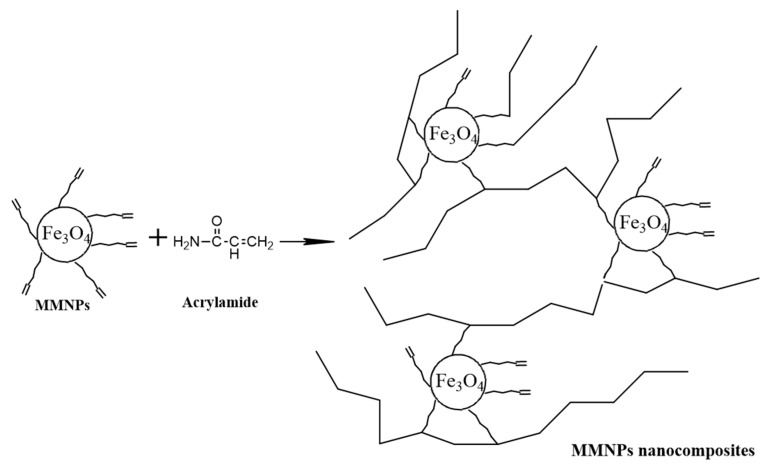
Preparation of polyacrylamide (PAA)/modified magnetic nanoparticles (MMNPs) nanocomposites.

Hydrogels were obtained by free-radical polymerization of acrylamide and MMNPs in aqueous solution (Figure 2). Briefly, various ratios between acrylamide monomer and MMNPs (90/10; 80/20; 70/30; 60/40 and 50/50 *w/w*) were prepared. The MMNPs were dispersed in water by sonication and added in a mixture of 15 wt. % aqueous acrylamide solution and initiator (potassium persulfate). The ratio between organic phase (acrylamide) and MMNPs was varied in order to enhance the mechanical properties of the hydrogels. The nanocomposite hydrogel samples were added in circular glass matrix and put at 60 °C for 24 h. Finally, the nanocomposite hydrogel samples were removed from the glass matrix and immersed in distilled water for 5 days to remove residual monomer and final purification. Hydrogels were cut as disks for further mechanical investigations (rheological measurements).

### 2.5. Swelling Measurements

Swelling behavior of the hydrogels was performed in saline solution at 37 °C. The weight changes of the hydrogels were recorded at regular time intervals during swelling. The swelling degree of the hydrogels was determined according to the following equation [42,43]:

(1) $$SD=\frac{W_{t}-W_{o}}{W_{0}}\cdot 100,$$

where W and W_0_ denote the weight of the wet hydrogel at a predetermined time and the weight of the dry sample, respectively. The equilibrium swelling degrees (ESD) were measured until the weight of the swollen hydrogels was constant. At least three swelling measurements were performed for each hydrogel sample and the mean values were reported.

Swelling kinetics. The dynamics of the water sorption process was studied by monitoring the saline solution absorption by the hydrogels at different time intervals. For diffusion kinetic analysis, the swelling results were used only up to 60% of the swelling curves. Fick’s equation was used [42,43,44,45,46,47,48,49]:

(2) $$f=k\cdot t^{n},$$

where f is the fractional water uptake, k is a constant, t is swelling time and n is the swelling coefficient that indicates whether diffusion or relaxation controls the swelling process. The fractional water content f is M_t_/M_n_ where M_t_ is the mass of water in the hydrogel at time t, and M_n_ is the mass of the water at equilibrium.

### 2.6. Characterization Methods

FTIR analysis. FTIR spectra of native magnetite and 3-TPM modified magnetite were recorded on a Bruker Vertex 70 FT-IR spectrophotometer with attenuated total reflectance (ATR) accessory with 32 scans and 4 cm^−^^1^ resolution in mid-IR region.

XPS analysis. The X-ray photoelectron spectroscopy spectra for magnetite and modified magnetite were recorded to evaluate the covalent bonding of silane modifying agent. The spectra were recorded on a K-Alpha instrument from Thermo Scientific, using a monochromated Al Kα source (1486.6 eV), at a pressure of 2 × 10^−9^ mbar.

#### 2.6.1. Evaluation of the Rheological Properties for the Nanocomposite Hydrogels

Rheological tests were performed with a rotational rheometer Kinexus Pro, Malvern Instruments, and a temperature control unit. In oscillating mode, a parallel plate and a geometric measuring system were used, and the gap was set according to the force value. The tests were performed on samples of 20 mm diameter with parallel plate geometry in a frequency range 1 to 30 Hz.

#### 2.6.2. Magnetic Properties by Vibrating Sample Magnetometry (VSM)

Vibrating sample magnetometry (LakeShore 7404-s VSM) was used in order to investigate the magnetic behavior of the hydrogels. Hysteresis loops were recorded at room temperature with an applied field up to 15 kOe, increments of 200 Oe and ramp rate of 20 Oe/s.

#### 2.6.3. Morphological Characterization by Scanning Electron Microscopy (SEM) and Transmission Electron Microscopy (TEM)

The microstructure of the samples was analyzed by Scanning Electron Microscopy (SEM) using a Quanta Inspect F50, with a field emission gun (FEG) having 1.2 nm resolution and an energy dispersive X-ray spectrometer (EDXS) having 133 eV resolution at MnKα. Morphology, geometrical evaluation (size and shape) of nanostructural characteristics and the crystalline structure of the samples were investigated by high-resolution transmission electron microscopy (HR-TEM) and selected area electron diffraction (SAED) using a TECNAI F30 G2 S-TWIN microscope operated at 300 kV with energy dispersive X-ray analysis (EDAX) facility.

## 3. Results and Discussion

### 3.1. Swelling Measurements

**Figure 3:**
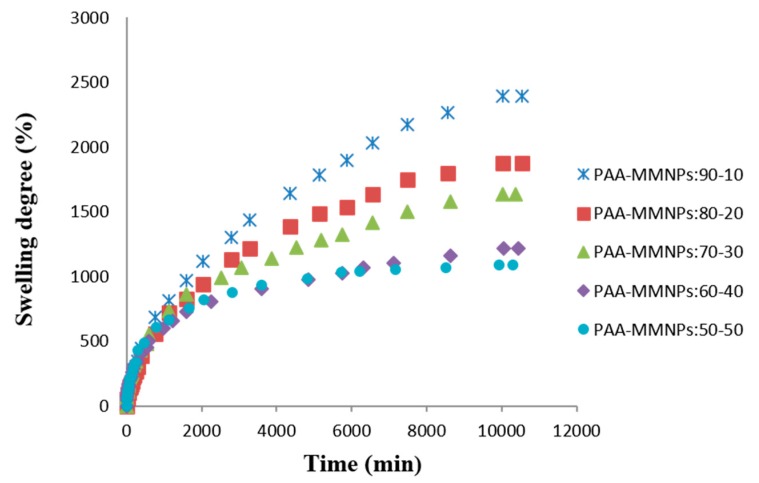
Swelling degree versus time in saline solution at 37 °C for PAA/MMNPs hydrogels.

The most important property of a hydrogel is its ability to absorb and hold an amount of solvent in its network structure. The equilibrium swelling of a hydrogel is a result of the balance of osmotic forces determined by the affinity to the solvent and network elasticity. Hydrogel properties depend strongly on the degree of cross-linking, the chemical composition of the polymer chains, and the interactions of the network and surrounding liquid. Figure 3 shows the water swelling behavior of the PAA/MMNPs hydrogels. The swelling curves show a decreasing trend of swelling degree with the increase of the modified magnetite nanoparticles content (Figure 3). These results are sustained by the fact that a higher amount of MMNPs lead to a higher crosslinking density. The crosslinking of the hydrogel comes from the reaction between the double bonds from NPs surface and the double bonds of the acrylamide monomer without the adding of any other crosslinker. 

Next, the swelling mechanism is evaluated by Equation (2). Here, by plotting ln f versus ln t, we may calculate the swelling coefficient n as the slope of the linear graph. It is known that the swelling process could be controlled by a Fickian-type mechanism, by relaxation of the chain or by both mechanisms depending on the composition. The values of n were below 0.5 for 2 samples (PAA/MMNPs 70/30, 60/40 ratio), which means a diffusion-controlled process (Fickian mechanism). The other three nanocomposite samples (PAA/MMNPs, 90/10, 80/20 and 50/50 ratio) are governed by a diffusion swelling coefficient with values above 0.5 and a water molecules transport model, done by chain relaxation [50,51]. These data are shown in Table 1.

### 3.2. FTIR Analysis

**Figure 4:**
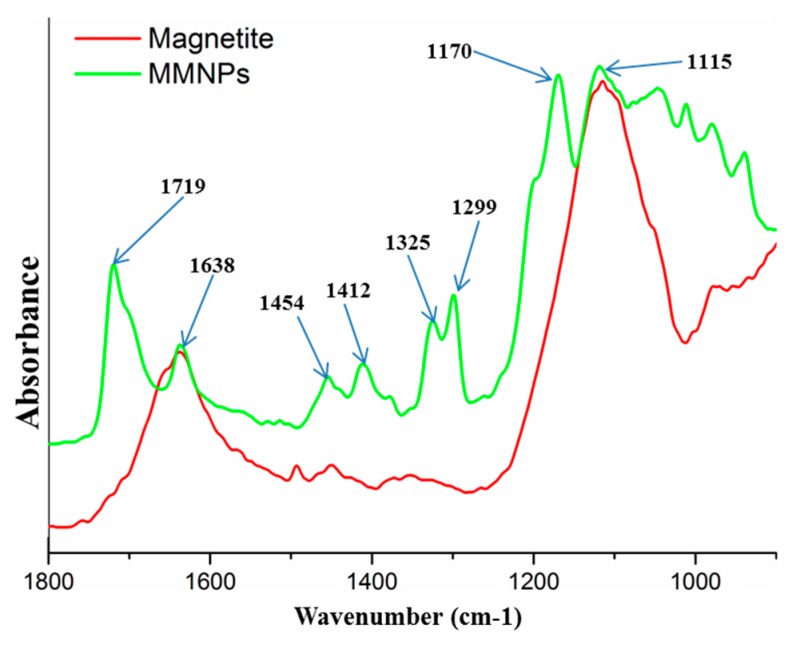
FTIR spectra for magnetite and double bond functionalized magnetite nanoparticles.

The modification of magnetite nanoparticles with 3-TPM was proved by FTIR investigation (Figure 4). FTIR spectrum of modified magnetite shows several new peaks specific to organic modifier 3-TPM. Therefore, the peak at 1170 cm^−^^1^ can be assigned to stretching vibration of ester bonds; peaks at 1299 cm^−^^1^ and 1325 cm^−^^1^ can be assigned to the stretching vibration of -Si-methylene- from the internal structure of modifier agent; peaks at 1454 cm^−^^1^ and 1412 cm^−^^1^ can be assigned to the bending vibration of methyl and methylene groups from the internal structure of the modifier agent; the peak at 1638 cm^−^^1^ is specific to the stretching vibration of –C=C– from the internal structure of the modifier agent; the peak at 1719 cm^−^^1^ is specific to the stretching vibration of carbonyl –C=O from the internal structure of the modifier agent [52]. Considering all of the attributed peaks, FTIR analysis was a very useful tool to evidence the modification of the magnetite nanoparticles with double bonds.

### 3.3. XPS Analysis

**Figure 5:**
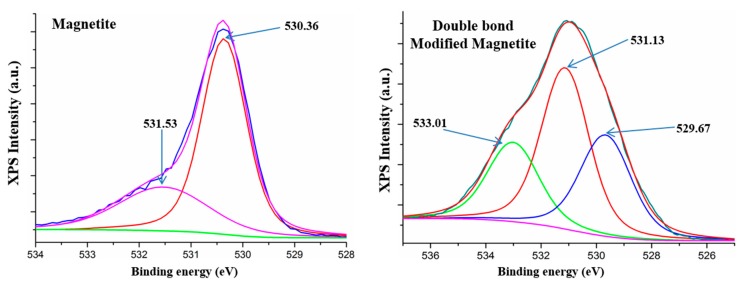
XPS spectra of magnetite and double bond modified magnetite.

XPS analysis for both magnetite and double bond modified magnetite was carried out in order to reveal the interstitial organic/inorganic character of new generated magnetite lattice. The results for surface modification are well correlated with the reaction mechanism and morphological results. There is an increasing of C1s in the elemental composition up to the main elemental percent, due to the modification on the surface of magnetite nanoparticles. Figure 5 highlights the high resolution spectra of the O1s species from crude magnetite with two deconvoluted peaks, the first centered at 530.35 eV, which can be attributed to O-Fe in magnetite phase [53], and the second centered at 531.01 eV, probably corresponding to the hydroxyl bonding within magnetite lattice. Furthermore, Figure 5 reveals the high magnification spectra of O1s species for functionalized magnetite nanoparticles with three secondary deconvoluted peaks. The two O1s peaks at 529.67 eV and 531.13 eV can be attributed to the crude magnetite structure and the new peak centered at 533.01 eV can be attributed to a Si-O new formed species by covalent bonding of silane with magnetite hydroxyl groups [22].

### 3.4. Evaluation of the Rheological Properties for the Nanocomposite Hydrogels

**Figure 6:**
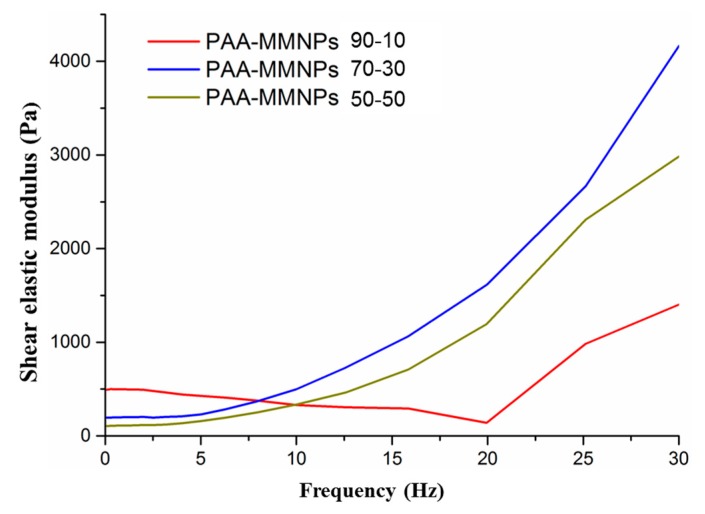
Elastic modulus G’ versus frequency.

Rheological behavior of novel nanocomposites was performed on swollen samples in aqueous NaCl 0.9 wt% solution at swelling equilibrium. The investigation involves the stress optimization in order to maintain a linear viscoelastic domain and samples to be dependent only on frequency and not on the applied stress. The elastic modulus for nanocomposite with 10% modified magnetite nanoparticles showed a unique behavior with significant differences, as compared to other samples. Figure 6 reveals a slow decreasing elastic of the modulus G’ up to 20 Hz, followed by a fast increasing until 30 Hz for the sample with 90% PAA and 10% modified magnetite nanoparticles. This behavior can be explained by a low amount of modified magnetite nanoparticles, which act as a crosslinking agent. The low amount of inorganic modified agent does not allow the specific elastic network to adapt to environmental mechanical changes [22]. The nanocomposite samples with a higher amount of modified magnetite nanoparticles (30%, 50%) showed a different specific elastic behavior with frequency variation, presenting a constant elastic modulus increasing from 1Hz up to 30 Hz. The specific elastic behavior allows for the environmental changes, due to the formation of elastically active chains by bridging multiple surrounding chains with various lengths. In the case of 30% modified magnetite nanoparticles, the elastic modulus exhibited higher values over the frequency range. This is probably due to the nanoparticles concentration that is optimal for a good dispersion into polymer matrix. In the case of the 50% modified magnetite nanoparticles, the elastic modulus showed lower values, probably due to a lower dispersion in the matrix, with significant influences on the segmental mobility of the 3D network.

### 3.5. Magnetic Properties by Vibrating Sample Magnetometry (VSM)

**Figure 7:**
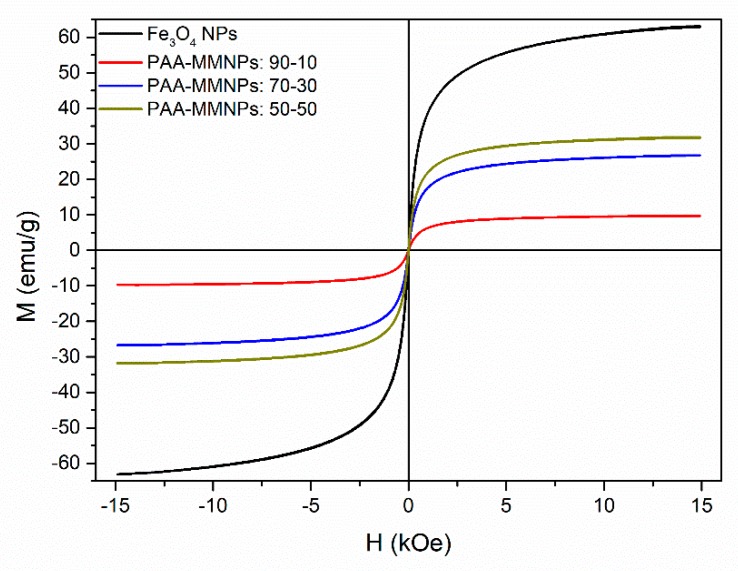
Vibrating sample magnetometry (VSM) magnetization curves of the Fe_3_O_4_ nanoparticles and the nanocomposites hydrogels.

The magnetic properties of the magnetic iron oxide nanoparticles (Fe_3_O_4_ NPs) and of the hydrogels were investigated by vibrating sample magnetometry (VSM) at room temperature. In Figure 7, the magnetic hysteresis loops that are characteristic of superparamagnetic behavior can be observed for all of the samples, due to the presence of the magnetite nanoparticles. Superparamagnetism is the responsiveness to an applied magnetic field without retaining any magnetism after removal of the applied magnetic field. The measured saturation magnetization (Ms) of the Fe_3_O_4_ NPs is 63.128 emu/g. For PAA-MMNPs 90:10, the saturation magnetization was found at 9.74 emu/g, the lowest measured saturation of the hydrogels. The saturation magnetization for the PAA-MMNPs 70:30 was found at 26.73 emu/g and the highest saturation magnetization was at 31.88 emu/g, corresponding to the PAA-MMNPs 50:50, the hydrogel with the highest concentration (50%) of MMNPs. These results show that the magnetization of the hydrogels increases with the increase of the concentration of MMNPs present in the hydrogels.

### 3.6. Morphological Characterization by SEM and TEM

#### 3.6.1. SEM Analysis

**Figure 8:**
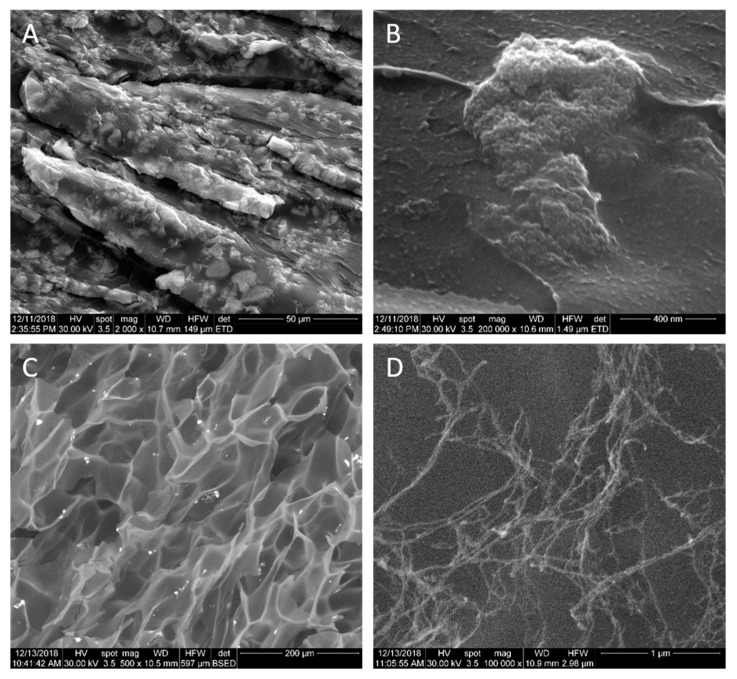
SEM micrographs of PAA-MMNPs 90:10 block hydrogel (**A**,**B**) and lyophilized PAA-MMNPs 90:10 hydrogel (**C**,**D**).

**Figure 9:**
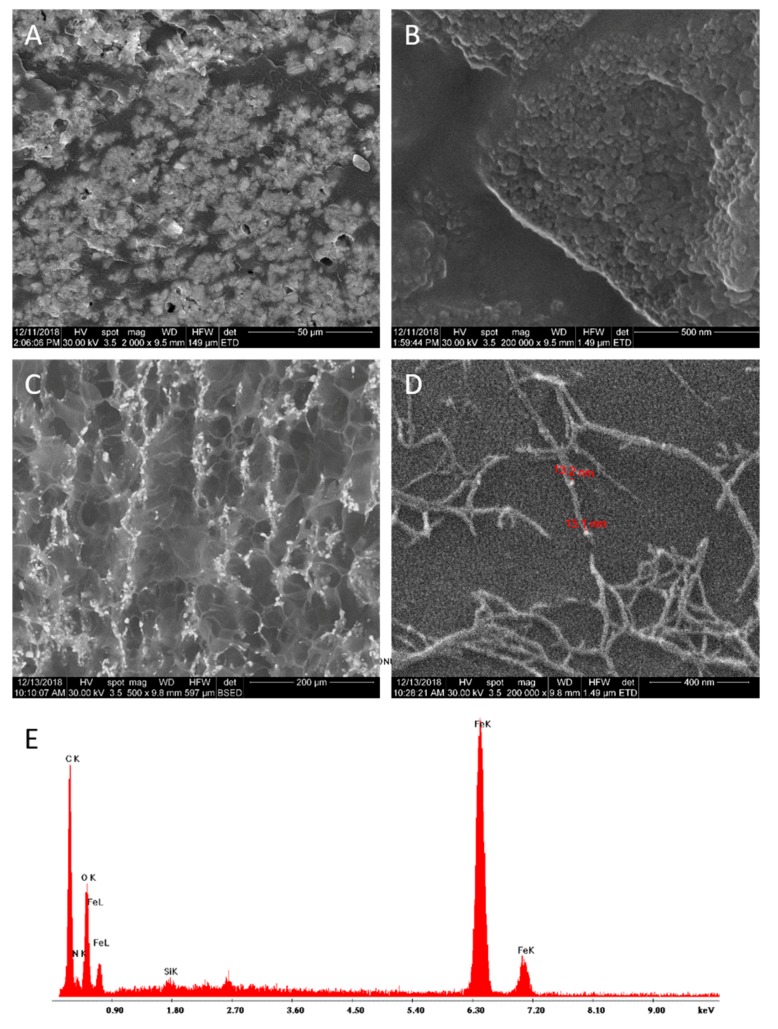
SEM micrographs of PAA-MMNPs 50:50 hydrogel (**A**,**B**).and lyophilized PAA-MMNPs 50:50 hydrogel (**C**,**D**); Energy dispersive X-ray (EDX) spectrum (**E**).

The microstructure of the PAA-MMNPs hydrogels was studied by SEM in cross-section and the results are shown in Figure 8 (PAA-MMNPs 90:10) and Figure 9 (PAA-MMNPs 50:50). The image in Figure 8A, (magnification ×2.000) shows submicronic areas of bright contrast (functionalized magnetite aggregates) evenly distributed in a dark contrast PAA matrix. At higher magnifications (×200.000, Figure 8B) it can be observed that the areas of bright contrast are aggregates of MMNPs. Also, the image shows that the modified Fe_3_O_4_ nanoparticles showed a good distribution in the polymer matrix by the presence of areas with high dispersed MMNPs and areas with local agglomeration of MMNPs. However, even the local agglomerations revealed that the modified magnetite nanoparticles (MMNPs) seem to be addressed by the polymer polyacrylamide matrix due to the effect of the crosslinking agent of the MMNPs (Figure 8A,B). Thus, the polymer matrix covering the MMNPs is chemically linked by the MMNPs and the whole ensemble displays a crosslinked network-like architecture.

Figure 8C is a SEM backscattered electron image at a smaller magnification (×500), showing small MMNPs agglomerates (white spots) uniformly dispersed on a mesh of micro-pores. Figure 8D is a detail (×100.000 magnification) of a nano-size area from the central zone in Figure 8C, showing a nanostructure of the lyophilized hydrogel as fibrils having evenly incorporated MMNPs. The crosslinked network-like ensemble generated by the MMNPs is better highlighted by the lyophilized samples (Figure 8C,D). The fibrils revealed branches-like structures which are extending on the sample surface and evenly through the sample internal structure. The branched-like structures exhibited MMNPs linked to each other by the polymer matrix and serve as the basis of the crosslinked network-like ensemble.

The SEM image in Figure 9A (magnification ×2.000) shows a higher density in MMNPs clusters for the PAA-MMNPs 50:50 hydrogel due to the higher amount of modified magnetite, in comparison to the PAA-MMNPs 90:10 hydrogel from Figure 8A. Detail from Figure 9A is shown in Figure 9B (magnification ×200.000), proving that the clusters are made of nanoparticles. The polymeric matrix is not homogenous, due to the fact that it has smaller nanoparticle aggregates embedded. The cross-section of the lyophilized hydrogel shows microsize pores, with chains of MMNPs clusters, which seem to be located especially on the pore walls. At higher magnifications (Figure 8D), it can be observed that there are also nano-size areas having the same fibrils with branches-like structures with incorporated MMNPs. A very interesting result of the lyophilized sample of both PAA-MMNPs 50:50 and PAA-MMNPs 90:10 (Figure 8C,D and Figure 9C,D) showed less local MMNPs agglomeration with respect to un-lyophilized samples. This behavior can be explained by the lyophilization procedure. During the process, the polymer matrix between MMNPs swells and the space grows between them. Furthermore, the sublimation phenomenon leads to a rearrangement of the structure with the display of the MMNPs in the pore walls and fragmentation of the local agglomerates.

The EDXS spectrum (Figure 9E), acquired on a large area of the PAA-MMNPs 50:50 hydrogel surface and shows the presence in the sample of the elements Fe and O (from Fe_3_O_4_ NPs), C, N and Si (from 3-TPM and PAA).

#### 3.6.2. TEM Analysis

The morphology and nanostructural characteristics of magnetic nanoparticles (MNPs), modified magnetic nanoparticles (MMNPs) and of polyacrylamide modified magnetic nanoparticles (PAA-MMNPs) hydrogels were analyzed by TEM, selected area electron diffraction (SAED) and high resolution electron microscopy (HR-TEM).

#### 3.6.3. TEM Analysis for Magnetite Nanoparticles (MNPS) and Modified Magnetite Nanoparticles (MMNPs)

**Figure 10:**
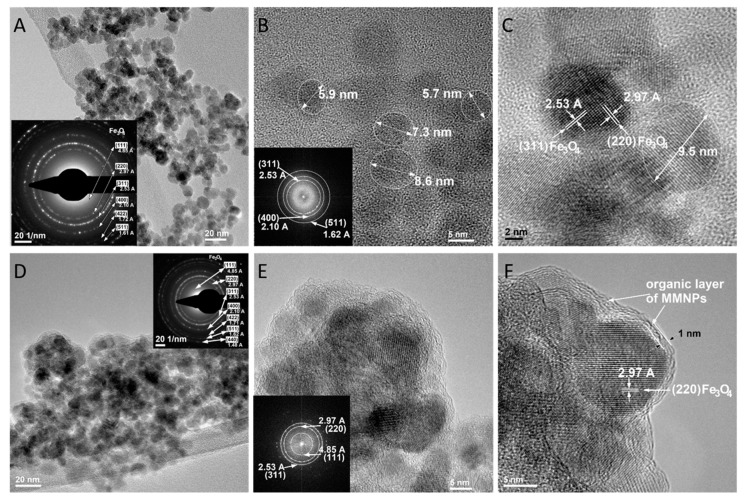
TEM images on Fe_3_O_4_ nanoparticles (**A**–**C**) and modified F_3_O_4_ nanoparticles (**D**–**F**).

Figure 10A–C are TEM micrographs of the MNPs. The bright field TEM image (Figure 10A) shows that the magnetic Fe_3_O_4_ nanoparticles are nearly spherical with diameters between 5 and 12 nm. The SAED pattern (inset of Figure 10A) of MNPs exhibits a typical face centered cubic (fcc) crystalline structure. The lattice spacing measured based on the diffractions rings is in accordance with the standard lattice spacing of Fe_3_O_4_ from the Powder Diffraction File (PDF) database (ICCD file no. 04-002-5683). The HRTEM images of MNPs (Figure 10B,C) clearly show the single crystallinity of Fe_3_O_4_ nanoparticles. The interplanar distances measured from the adjacent lattice fringes with Fast Fourier Transform (FFT) (inset of Figure 10B) are 2.53 Å, 2.10 Å and 1.62 Å, corresponding to (311), (400) and (511) crystalline family planes of Fe_3_O_4_ with crystalline structure, according to the PDF database. Nanocrystalline particles with diameter size between 5.7 and 8.6 nm are highlighted in Figure 10B. In the HRTEM image from Figure 10C it clearly shows the crystalline planes with 2.97 Å and 2.53 Å measured interplanar distances corresponding to crystalline family planes with (220) and (311) Miller indices. 

The TEM results of MMNPs are presented in Figure 10D,E,F. According to Figure 10D, modified Fe_3_O_4_ nanoparticles still keep the morphological properties of Fe_3_O_4_ nanoparticles. According to HRTEM images (Figure 10E,F), morphological and nanocrystalline properties of Fe_3_O_4_ nanoparticles are maintained, but it is clearly shown that the nanoscale Fe_3_O_4_ nanoparticles are modified in the MMNPs sample, because of the organic layer surrounding the Fe_3_O_4_ nanoparticles (highlighted in Figure 10F). The tailoring of magnetite nanoparticles by chemically functionalization with silane 3-TPM revealed by physico-chemical X-photoelectron spectroscopy is also sustained by the morphological characterization by TEM. The high magnification Figure 10E,F exhibits a less ordered organic layer consisted by silane 3-TPM, which addresses the magnetite nanoparticles. However, the surrounding organic layer displayed a specific order and arrangement structure, which will be further discussed. The Figure 10D revealed an overview result with a considering functionalization of the whole nanoparticles and not as an isolated modification. Thus, the magnetic nanoparticles were tailored with double bonds by the presence of the silane structure (Figure 1).

#### 3.6.4. TEM Analysis of Polyacrylamide-MMNPs Nanocomposite Hydrogels

**Figure 11:**
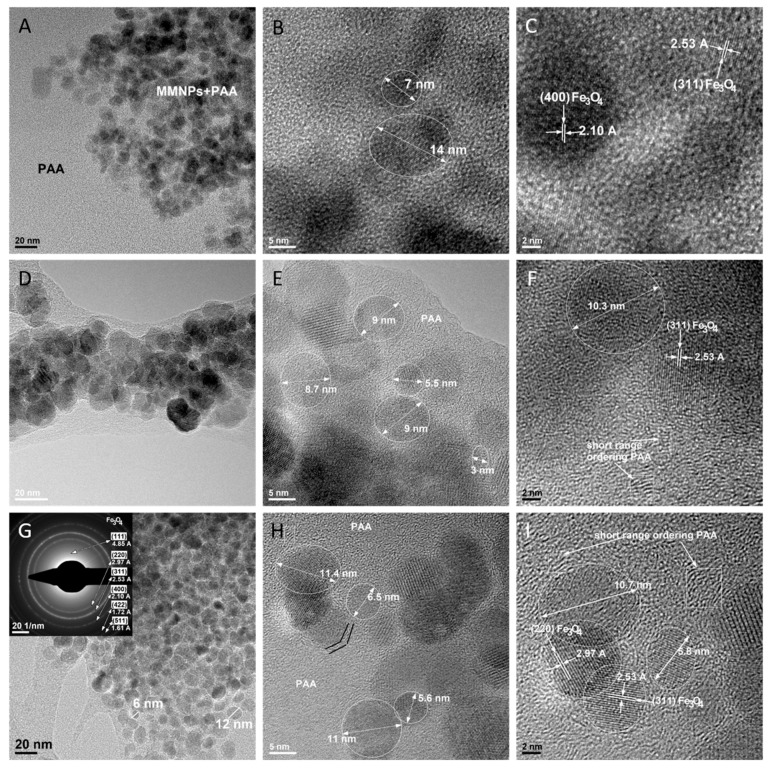
Bright field (BF)-TEM and HR-TEM images on PAA-MMNPs 90:10 (**A**–**C**); PAA-MMNPs 70:30 (**D**–**F**) and on PAA-MMNPs 50:50 (**G**–**I**).

The bright field TEM (BF-TEM) images from Figure 11A, 10D and 10G are results from PAA-MMNPs 90:10, PAA-MMNPs 70:30 and PAA-MMNPs 50:50 samples. These images show that all of the hydrogels have a similar morphology and nanostructure. The overview images (Figure 11A,D,G) revealed an expected decreasing of polymer matrix area with the increasing of MMNPs amount. All of the samples have isolated and local agglomerated magnetic Fe_3_O_4_ nanoparticles embedded within a polymer matrix. By comparing the BF-TEM images from PAA-MMNPs (Figure 11A,D,G) with the BF-TEM images from MNPs (Figure 10A) and MMNPs (Figure 10D), it can be concluded that the shape and the dimensions of the embedded nanoparticles are kept in the same range. The SAED image (inset of Figure 11G) shows that the PAA-MMNPs hydrogels contain similar Fe_3_O_4_ nanoparticles, well crystallized, with the same lattice spacing measured on the SAED image from MNPs (inset of Figure 10A). The diffraction of the matrix was not observed in the SAED image (inset of Figure 11G), which is probably because the organic layer and the PAA matrix are not highly ordered and are displaying short ordering range. In order to observe the detailed structure of PAA-MMNPs hydrogels, HRTEM was employed. Figure 11B,C (from PAA-MMNPs: 90-10), Figure 11E,F (From PAA-MMNPs: 70-30) and Figure 11H,I (from PAA-MMNPs: 50-50) show that the nanoparticles are embedded within a polymer matrix with amorphous structure. The nanoparticles have a round shape with diameters between 5 and 14 nm. The MMNPs are well integrated into polymer matrix revealing a clear interaction between the two phases. The nature of the interaction was revealed by the HR-TEM images, which rarely highlighted the specific order and arrangement structure of the organic layer from MMNPs. This result can be explained by the surrounding organic layer being in a chemical reaction with the acrylamide monomer by the consumption of the silane double bonds. Thus, the MMNPs act as an inorganic cross-linker by becoming generators of bridges between polymeric chains and development of a hybrid network (Figure 2). Also, the HRTEM results show that the nanoparticles are nanocrystals, disclosing the crystalline planes (220) and (311) of magnetite with 2.97 Å and 2.53 Å, respectively, which are characteristic interplanar distances. Furthermore, the HRTEM images also reveal a short ordering range in the matrix besides the amorphous phase, highlighted by squares (Figure 11F for PAA-MMNPs: 70-30 and Figure 11I for PAA-MMNPs: 50-50), which shows the structural arrangement of the polymer macromolecular chains compared with inorganic ordered magnetite nanoparticles.

## 4. Conclusions

This study provides a comprehensive approach in the wide field of polymer nanocomposite materials. A new hybrid polymer network was successfully developed by double bond modified magnetic nanoparticles, using polyacrylamide as the crosslinked network structure, thereby overcoming the limitation of traditional organically crosslinkers. Functionalization of magnetic nanoparticles with the double bond was monitored by physico-chemical investigations. The details of the microarchitecture were shown by modern morphological characterization techniques, highlighting the nature of the interaction between the organic and inorganic phases. Furthermore, the obtained nanocomposite hydrogels may have an efficient applicability in the soft tissue engineering field, in the form of repairing scaffolds, due to their mechanical and magnetization behavior that can stimulate tissue regeneration.

## Figures and Tables

**Table 1:** The swelling diffusion coefficient and the regression model-R^2^.

Parameters/ Composition	PAA/MMNPs 90:10	PAA/MMNPs 80:20	PAA/MMNPs 70:30	PAA/MMNPs 60:40	PAA/MMNPs 50:50
n	0.6044	0.5816	0.4400	0.4818	0.5465
R^2^	0.9980	0.9979	0.9985	0.9981	0.9939
